# Plasma complex lipids in relation to cortical thickness and brain volumes: results from the population-based Rhineland study

**DOI:** 10.1186/s12944-026-02930-5

**Published:** 2026-03-19

**Authors:** Elvire N. Landstra, Valerie Lohner, Juliana F. Tavares, Kersten Diers, Alexandra Koch, Monique M.B. Breteler

**Affiliations:** 1 Population Health Sciences, German Centre for Neurodegenerative Diseases (DZNE), Venusberg-Campus 1, Building 99, Bonn, 53127 Germany; 2 AI in Medical Imaging, German Centre for Neurodegenerative Diseases (DZNE), Bonn, Germany; 3 Institute for Medical Biometry, Informatics and Epidemiology (IMBIE), Faculty of Medicine, University of Bonn, Bonn, Germany

**Keywords:** Complex lipids, Fatty acids, Sphingolipids, Neutral lipids, Phospholipids, Population health, Brain imaging

## Abstract

**Background:**

The brain is man’s second-fattiest organ and shows a remarkable lipid diversity. Despite recent advances in lipidomics, much remains unknown about the connection between blood lipids and the brain. Therefore, we investigated the relation between circulating lipid levels and cortical thickness and brain volumes.

**Methods:**

We related blood levels of ~ 1000 lipid species and fatty acid composite measures to cortical thickness, and total brain, white matter and grey matter volume in the Rhineland Study (*n* = 3,248) using linear regression models, adjusting for sex, age and low-density and high-density lipoprotein cholesterol. To assess region-specific effects in the relation between lipids and cortical thickness, we performed a cortex-wide vertex-based analysis on our top hits.

**Results:**

Chain length and degree of saturation influenced a lipid’s effect across different markers of brain health (cortical thickness, brain volumes). Specifically, odd-chained and 18-carbon carrying lipids were mostly associated with a thicker cortex and larger total, grey and white matter volume, whereas 16-carbon carrying and highly unsaturated (> 4 bonds) lipids were associated with a thinner cortex and smaller brain volumes. Furthermore, we found that lipids were mainly associated with regions outside of the pre-frontal cortex and related to the two hemispheres asymmetrically.

**Conclusions:**

Investigation of within-class relative lipid concentrations suggested that higher levels of odd-chained and 18-carbon carrying lipids and lower levels of polyunsaturated and 16-carbon carrying lipids could be crucial for brain structure. Our research highlights potential targets to improve brain health and underscores that lipid profiles, rather than total lipid concentrations, are critical.

**Supplementary Information:**

The online version contains supplementary material available at 10.1186/s12944-026-02930-5.

## Background

The brain is the second fattiest organ of the human body, with lipids constituting around 50% of its dry weight [1]. Besides a high overall lipid content, the brain also contains a remarkable diversity of lipid classes and species, which are crucial for synaptogenesis, the formation of biomembranes, signal transduction, and as energy source [2]. Hence, lipids have become a subject of increasing interest in studying neurodegenerative diseases [1, 3–5].

Recent advances in lipidomics allow for large-scale detection of a wide range of lipids with varying functions. Based on their basic biochemical structure, namely the number of fatty acid tails and their headgroup, lipid species can be grouped into distinct lipid classes [6]. The biological importance of different lipid classes and their ratios to each other has been demonstrated in multiple settings. For example, higher levels of sphingomyelins and ceramides have been linked to pathologies, including Alzheimer’s disease (AD) and cardiovascular disease (CVD) [7–10]. Diacylglycerols and monoacylglycerols were found to be elevated in the plasma and brain of patients with mild cognitive impairment [11]. Other lipids have been suggested to have beneficial effects, such as some phosphatidylcholines [12]. Lastly, the balance between particular lipid classes is crucial for health too. The ratio between cholesteryl esters and free cholesterol is necessary for proper immune cell function [13], and the ratio between phosphatidylcholines and phosphatidylethanolamines is crucial for energy metabolism among others [14].

Besides lipid class, attributes such as fatty acid chain length and degree of saturation can be responsible for distinct effects across lipid species [12, 15–19]. Compared to their shorter-living counterparts and species, long-living humans and long-lived species were found to have lower blood levels of long-chained free fatty acids, particularly polyunsaturated fatty acids (PUFAs), possibly because this provides more resistance to oxidative stress [15, 20, 21]. Odd-chained fatty acids have been consistently linked to a decreased risk of type 2 diabetes (DM2) [17, 22]. Furthermore, chain length and degree of saturation reportedly are important in the relations of phosphatidylethanolamines, diacylglycerols, triacylglycerols and cholesteryl esters with AD [19, 23], triacylglycerols with cortical thickness and longevity [12, 18], and both ceramides and free fatty acids with CVD and chronic obstructive pulmonary disease (COPD) [7, 24].

While specific lipid classes and characteristics have thus been associated with longevity and pathology [12, 17, 22], most research has focused on either traditional combined lipid measurements (e.g. total cholesterol) [25], only assessed a limited number of lipid species [7, 18, 26], or was restricted to case-control designs or specific populations [12, 15], such as people with AD [19, 27]. Furthermore, despite the brain containing a considerable number of lipids, the association between circulating lipids and the brain has not been extensively investigated. Because the plasma lipidome is the result of all physiological and pathological processes in the body, including the brain [15, 28], its profiling might yield a minimally invasive tool to discover biomarkers and provide mechanistical insights into brain physiology and neurodegenerative diseases. We therefore examined the relation between concentrations of ~ 1,000 circulating lipid species and specific imaging markers of brain health (cortical thickness, brain volumes) in a population-based study.

## Methods

### Study population

The current study is based on the first 5,000 consecutive participants of the Rhineland Study, an ongoing community-based cohort study in Bonn, Germany. This analysis is based on participants for whom data was collected between 2016 and 2021. To be eligible for participation, people have to be over 30 years old and reside in either of two geographically defined areas in Bonn, Germany. Participation is possible by invitation only with the sole exclusion criterion being inadequate command of the German language to provide informed consent. The study is carried out following the recommendations of the International Council for Harmonization Good Clinical Practice standards and approval to undertake the study was given by the Medical Ethics Committee of the University of Bonn. Written informed consent is obtained from all participants in accordance with the declaration of Helsinki.

The Rhineland Study employs a deep phenotyping approach and collects data on various measures, including brain MRI, genetics, metabolomics, and lipidomics.

### Brain MRI measurements

MRI data were acquired on 3 Tesla MRI scanners (MAGNETOM Prisma, Siemens Healthineers, Erlangen, Germany) equipped with an 80 mT/m gradient system and a 64-channel phased-array head-neck coil, as previously described [29]. Brain structure volumes (total brain volume, grey matter volume, white matter volume; in cm^3^) and cortical thickness (in mm) were obtained using the standard FreeSurfer (version 6.0) processing pipeline on T1-weighted magnetic resonance images [30, 31]. We used the estimated total intracranial volume generated by FreeSurfer as a proxy for head size [32]. Quality of the FreeSurfer outputs was assured by visual inspection of anatomical segmentation images. Based on this process, which has been described in detail elsewhere [33], data from some participants was excluded in the quality control.

### Lipids

Blood was withdrawn intravenously in the morning, after overnight fasting. We acquired both the absolute (nmol/mL) and relative (mol%) lipid concentrations in fasting plasma samples from the Metabolon Complex Lipids platform. Lipid extraction was performed with internal standards using an automated butanol/methanol (BUME) extraction according to the method of Lofgren et al. [34]. Run-day and inter-batch normalizations were performed to correct for batch effects. Absolute concentrations of lipids were calculated as a ratio of the peak intensity of the target compound to that of its corresponding internal standard of known concentration after background-subtraction. Relative concentrations (mol%) of lipid species were calculated as a proportion of the total concentration of the relevant class; fatty acid composite measures by the proportion of each class comprised by individual fatty acids; and class totals as the proportion of the total lipid concentration.

We identified almost 1,000 individual species, spanning 14 major lipid classes (Additional file 1). The phosphatidylethanolamine lipid class encompassed phosphatidylethanolamine esters, phosphatidylethanolamine ethers and phosphatidylethanolamine plasmalogens. Only lipid species with detectable levels in at least 10% of participants were included in the analysis (*n* = 944). We distinguished between species and fatty acid composite measures (Additional file 2). Briefly, species refer to lipids where we know the total number of carbons and double bonds and where up to two fatty acid tails are identified (e.g. MAG(18:3) and DAG(16:1/20:0)). For the three-tailed triacylgycerols only the total number of carbons and double bonds and information on one fatty acid tail was obtained (e.g. TAG50:3(FA20:3)). Fatty acid composite measures sum up all species of a specific length or degree of saturation. For example, TAG(FA12:0) reflects the concentration of all triaycylglycerol species with at least one fatty acid tail of 12 carbons and 0 double bonds.

### Covariates and descriptors

Covariates included sex (women/men), age (years), and clinically measured high-density lipoprotein cholesterol (HDL-C), and low-density lipoprotein cholesterol (LDL-C). Prevalent hypertension (no/yes, controlled/yes, uncontrolled/yes, unknown) combined self-reported hypertension, regular anti-hypertensive medication use, and mean diastolic and systolic blood pressure being high (> 90, > 140, respectively). Use of lipid-lowering medication was defined as the use of any lipid-modifying agents (ATC code C10, A10BH51). Body mass index (BMI) was calculated using weight and height measured at the study center.

### Statistical analysis

We modelled the brain outcomes as a function of the log-transformed, standardized lipid measurements in linear regression models on participants with complete data. We assessed the effect of both absolute (nmol) and relative (mol%) concentrations of lipids on brain outcomes, with the relative concentrations reflecting the lipid concentrations relative to the other measured lipids. All outcomes were scaled to facilitate comparisons. Hence, the results are depicted as difference in the SD of the respective brain outcome.

We ran unadjusted models before correcting for age, sex, and LDL-C and HDL-C levels. In the models with brain volumes as outcome, we additionally adjusted for estimated total intracranial volume. P-values were adjusted for multiple testing using the false-discovery-rate (FDR) method across all outcomes. An FDR corrected p-value of < 0.05 was considered significant.

To assess the relative importance of different lipid classes, we calculated the enrichment of classes over others as a function of the proportion of observed significant associations over the expected significant associations for the relevant class.

The population distribution of many of these lipids have not yet been determined. As statistical outliers in the data might reflect actual biological values, a priori excluding them would be inappropriate. However, to account for possible measurement errors and to assess the robustness of the results, we ran sensitivity analyses by excluding lipid values more than 5 SDs away from the mean on a per lipid basis. To investigate whether associations were influenced by the intake of lipid-lowering medication, we ran an additional sensitivity analysis excluding people on lipid-lowering medication.

To assess whether the observed effects of lipids on cortical thickness were uniform across or specific to certain brain regions, we performed cortex-wide vertex-based analyses. We did this for the relative concentrations of the 6 total fatty acid composite measures that were most strongly associated with cortical thickness based on their p-values after adjustment for sex, age, LDL-C, and HDL-C. Analyses were corrected for age, sex, and LDL-C and HDL-C levels. The cortical thickness maps were laid out over a group surface and smoothed with 10 mm full-width-half-maximum (FWHM) kernel. A permutation simulation cluster-wise correction based on 1,000 iterations and a cluster-forming threshold of *p* < 0.05 was carried out to adjust for multiple testing [35].

The vertex analysis was done in FreeSurfer (version 6.0) [31], all others analyses were performed in R version 4.2.1 [36].

## Results

**Fig. 1:**
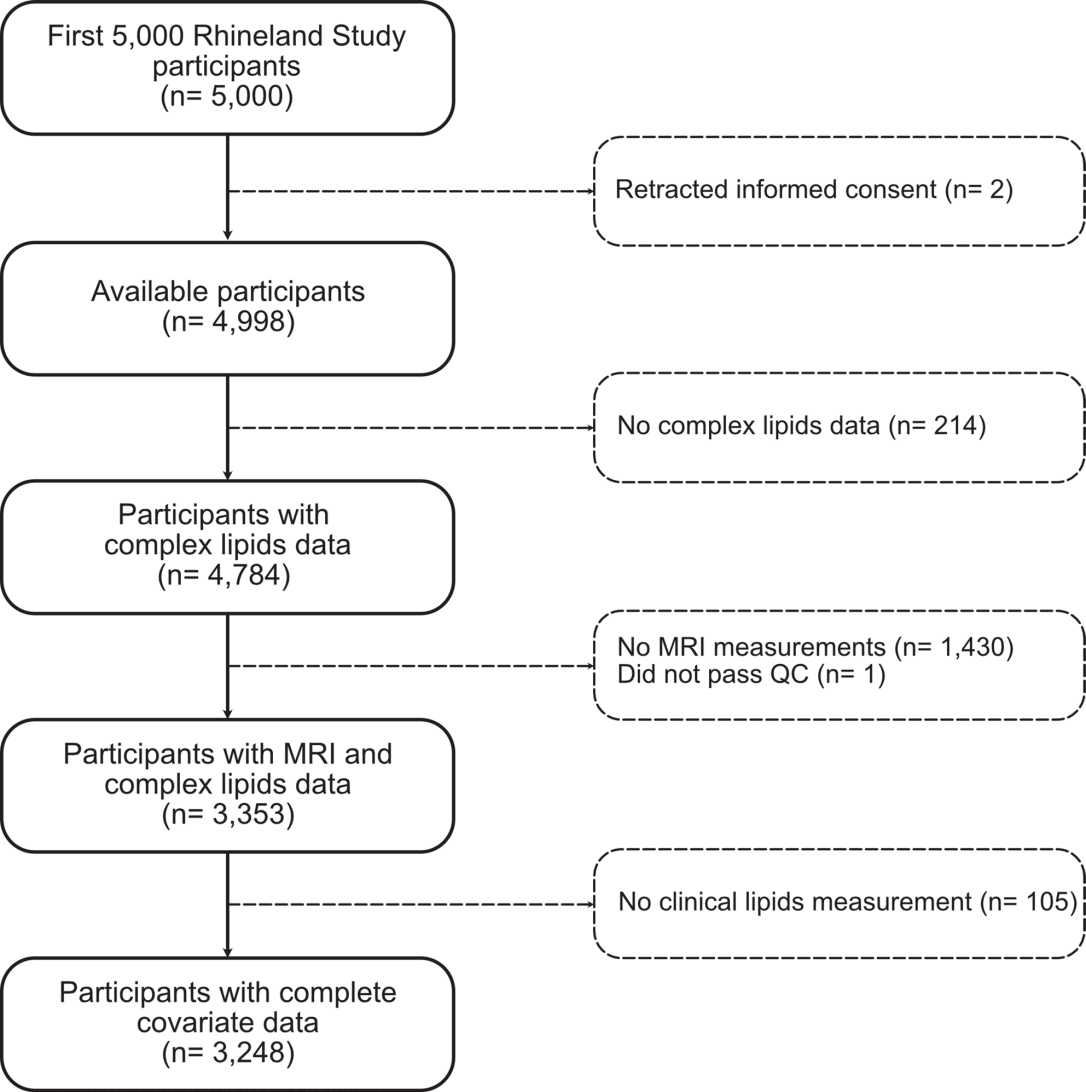
Flowchart of participant selection. A flowchart showing the selection criteria and the creation of the analysis sample

The current study was based on the first 5,000 consecutive Rhineland Study participants. We excluded people who had retracted their informed consent (*n* = 2), without sufficient plasma for lipidomics analysis (*n* = 214), without available MRI data (*n* = 1,430), and whose LDL-C and HDL-C concentrations were not measured (*n* = 106) (Fig. 1). This left an analytical sample of 3,248 participants with complete data. Included participants had a mean age of 54.2 years (± 14, range 30–95 years), were largely women (57%), and they had mostly obtained a high (55.4%) or medium high (42.3%) education level. The sample was relatively healthy with an average BMI of 25.6 (± 4.2) and only 10.9% of participants using lipid-lowering medication. The complete characteristics of the study participants and their lipid class concentrations can be found in Table 1 and Additional file 1, respectively.

**Table 1:** Overview of participant characteristics

Characteristic		Participants (*n* = 3,248)
Age (years (range; SD)		54 (30–95; 14)
Sex (N (%))	*Men*	1,384 (42.6)
	*Women*	1,864 (57.4)
Education level (N (%))	*Low*	53 (1.6)
	*Middle*	1,373 (42.3)
	*High*	1,801(55.4)
	*Unknown*	21 (0.6)
LDL-C (mg/dL (SD))		127.0 (35.7)
HDL-C (mg/dL (SD))		63.2 (17.9)
Body mass index (kg/m2 (SD))		25.6 (4.2)
Hypertension (N (%))	*No*	2,094 (64.3)
	*Yes*,* controlled*	488 (15.3)
	*Yes*,* uncontrolled*	611 (19.2)
	*Yes*,* unknown*	37 (1.2)
Use of lipid-lowering medication (N (%))		349 (10.9)
*Brain volumes*		
Total brain volume (cm^3^ (SD))		1,108.5 (117.6)
Grey matter volume (cm^3^ (SD))		625.3 (61.7)
White matter volume (cm^3^ (SD))		456.8 (58.6)
*Cortical thickness*		
Cortical thickness (mm (SD))		2.4 (0.1)

### There is substantial intraclass variability in the effects of lipid species on the brain

**Fig. 2:**
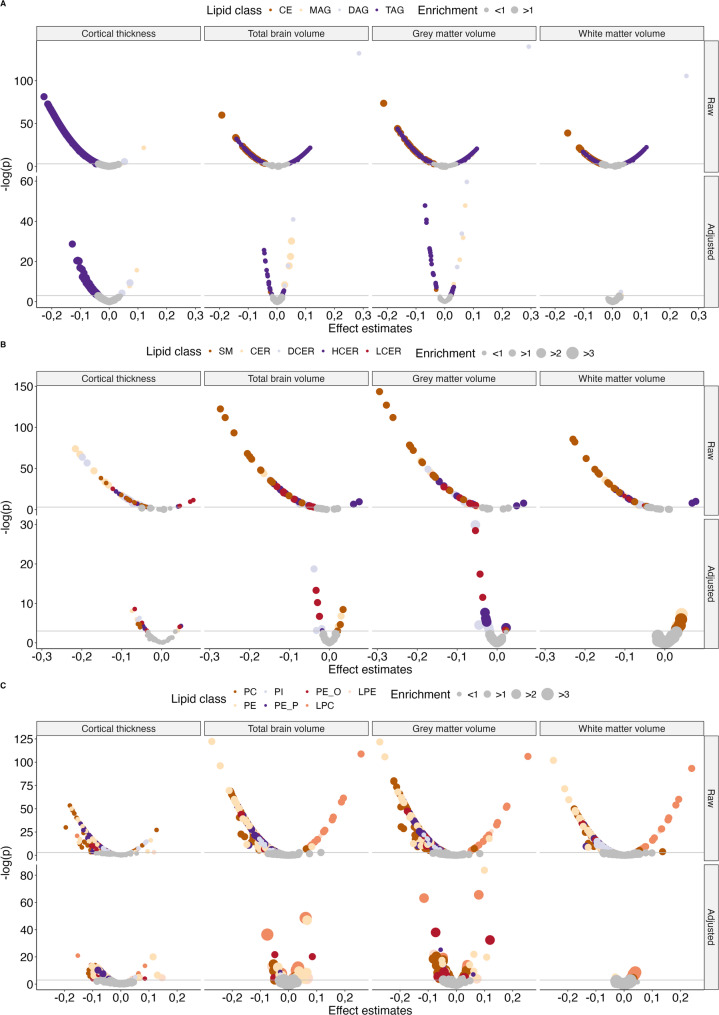
Volcano plots visualizing effect sizes (x-axis) against statistical significance (y-axis) for the associations between lipid species and cortical thickness and brain volumes. The plotted beta estimates (x-axis) against the log-transformed p-value (y-axis) for the raw and adjusted associations between brain imaging markers (columns) and absolute concentrations of lipid species, coloured by lipid class grouped into neutral (**A**), sphingo- (**B**) and phospholipids (**C**). The size of the dot indicates whether we find more or fewer significant associations within that lipid class than expected (enrichment). Non-significant estimates are coloured in grey. Adjusted estimates were adjusted for age, sex, low-density lipoprotein cholesterol (LDL-C) and high-density lipoprotein cholesterol (HDL-C) concentrations and estimated intracranial volume where applicable.

We found diverse effects for lipid species belonging to the same class or category. In all three categories and 14 classe*s* we observed lipid species related to either a thicker or thinner cortex and to larger or smaller brain volumes, both before (Fig. 2A) and after adjustment for sex, age, LDL-C, and HDL-C (Fig. 2B). For example, while the majority of triacylglycerols were associated with a larger total brain and grey matter volume after adjustment, the triacylglycerols that were associated with a smaller total brain and grey matter volume had substantially larger absolute effects (total brain volume: beta estimate (β); range: -0.05; 0.02, grey matter volume: β; range: -0.07; -0.03) after adjustment. Similarly, most phosphatidylethanolamines were weakly associated with a thinner cortex, but phosphatidylethanolamines that were associated with a thicker cortex, while fewer in number, had stronger effects (β; range: -0.10; 0.13). This intraclass variability became even more pronounced for associations between the relative species concentrations and brain outcomes (Additional file 3 and 4). All βs, confidence intervals (CIs) and adjusted p-values can be found in Additional file 5.

In our sensitivity analyses, we found that excluding outliers or people on lipid-lowering medication did not substantially alter the results (Additional file 5).

### Length and degree of saturation of the fatty acid tail determines the effect of a lipid

**Fig. 3:**
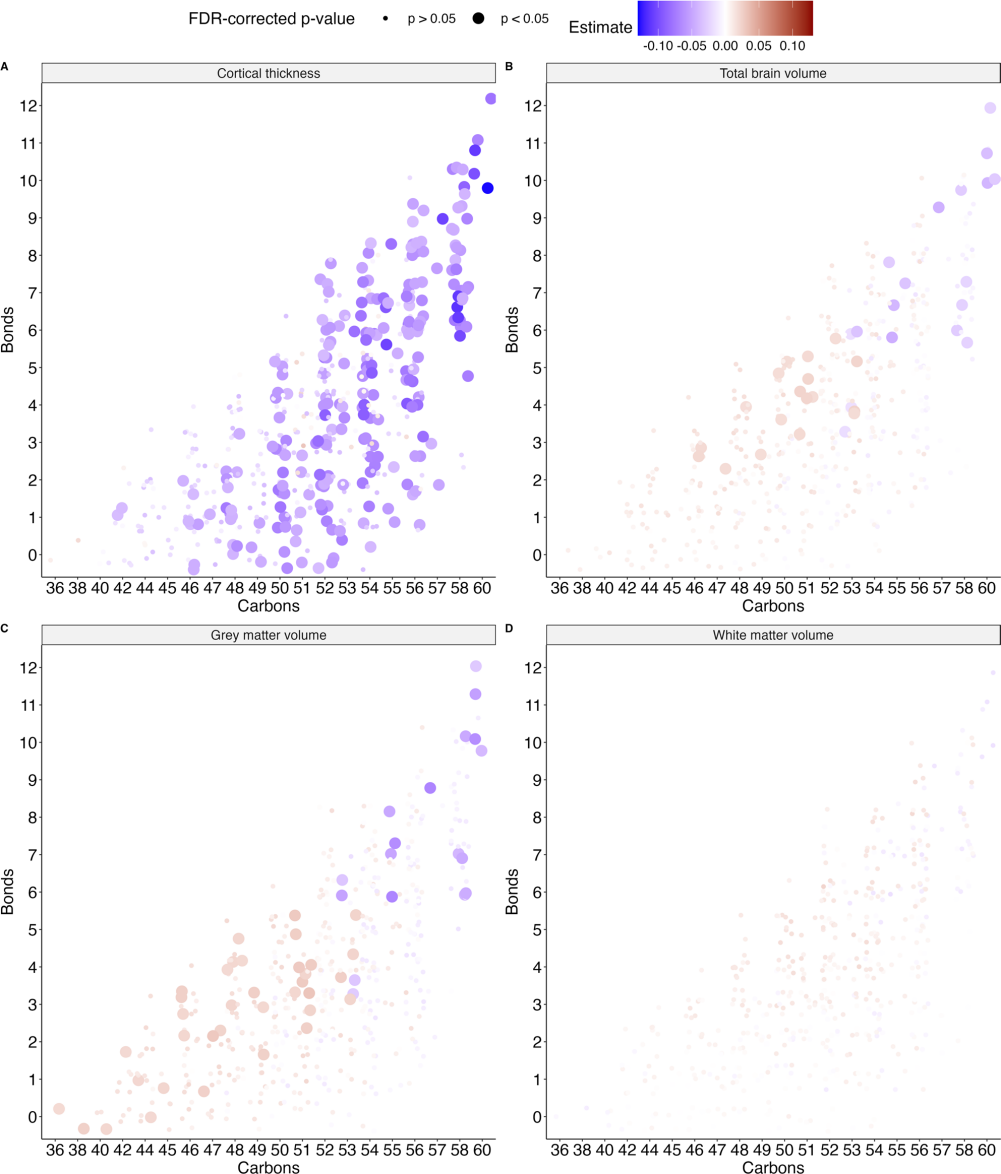
Associations between TAGs and cortical thickness and brain volumes. Beta estimates for the absolute concentrations of TAGs by the total number of double bonds (y-axis) and carbons (x-axis) for cortical thickness (**A**), total brain volume (**B**), grey matter volume (**C**) and white matter volume (**D**). FDR-corrected significant associations are indicated by size. Estimates were adjusted for age, sex, low-density lipoprotein cholesterol (LDL-C) and high-density lipoprotein cholesterol (HDL-C) concentrations and estimated intracranial volume where applicable

We investigated the intraclass variability further by focusing on different properties of the lipid tails. We first considered triacylglycerol species, as these are the only lipids with three fatty acid tails in our set and have previously been associated with cortical thickness [18]. We found that longer, more unsaturated triacylglycerols were more strongly associated with a thinner cortex and smaller total brain and grey matter volume after adjustment for sex, age, LDL-C, and HDL-C. On the other hand, shorter, less unsaturated species were associated with a larger total brain and grey matter volume (Fig. 3A and B).

**Fig. 4:**
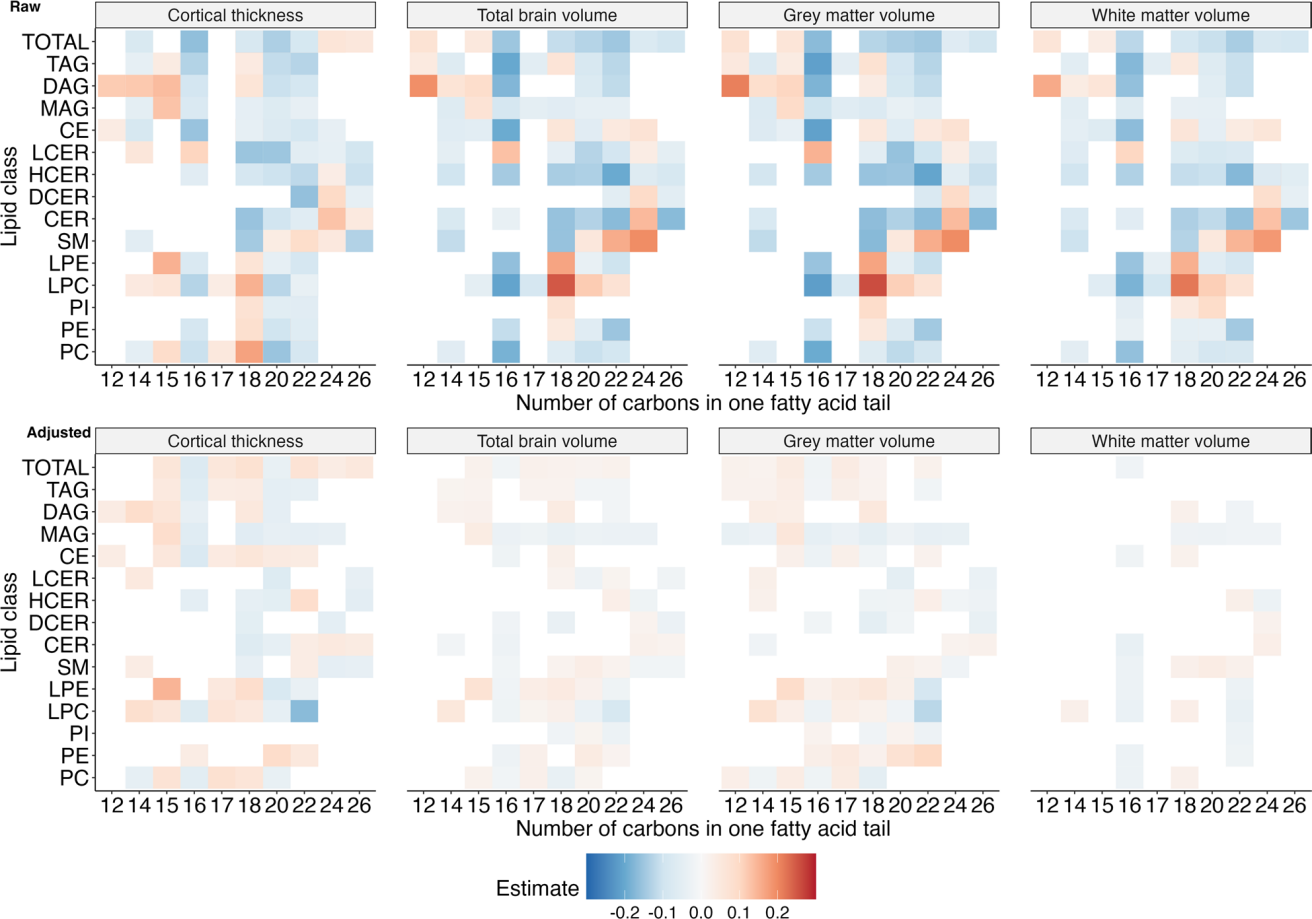
Associations between fatty acid composite measures and cortical thickness and brain volumes by length. Significant raw (**A**) and adjusted (**B**) beta estimates for the relative concentration of composite measures per lipid class (y-axis) by the number of carbons in the fatty acid tail (x-axis). Estimates were adjusted for age, sex, low-density lipoprotein cholesterol (LDL-C) and high-density lipoprotein cholesterol (HDL-C) concentrations and estimated intracranial volume where applicable

**Fig. 5:**
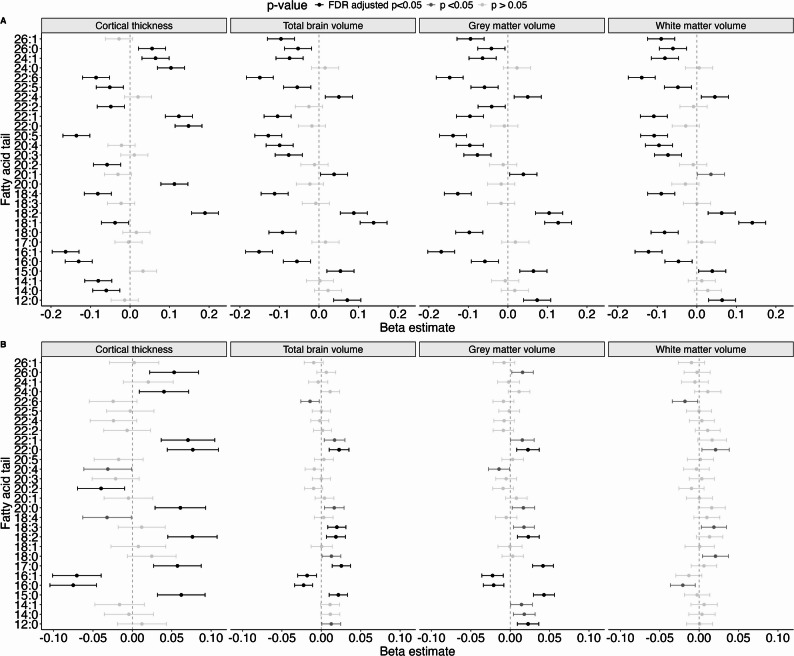
Associations between total fatty acid composition and cortical thickness and brain volumes. Forest plot showing the raw (**A**) and adjusted (**B**) beta estimates and 95% confidence intervals (x-axis) of the associations between the total relative fatty acid composite measures (y-axis), calculated across all lipid classes, and the different brain outcomes (columns). Estimates were adjusted for age, sex, low-density lipoprotein cholesterol (LDL-C) and high-density lipoprotein cholesterol (HDL-C) concentrations and estimated intracranial volume where applicable

Next, we investigated the fatty acid composite measures, which sum up the concentration of all possible fatty acid tails within a class, or across all classes. Therewith, we found that the length of a lipid’s fatty acid tail was important for its association with brain volumes and cortical thickness (Figs. 4 and 5).

Firstly, out of 25 16-carbon carrying fatty acid composite measures, the relative concentrations of 16 were significantly associated with cortical thickness, 18 with total brain volume, 15 with grey matter volume, and 6 with white matter volume after adjustment for sex, age, LDL-C, and HDL-C. Of these, the majority was associated with a thinner cortex (88%), and smaller total brain (83%), grey matter (73%), and white matter volume (100%). Our findings for total fatty acid composite measures confirmed these patterns (Fig. 5B). We found that higher relative concentrations of both total FA16:0 and FA16:1 were strongly associated with a thinner cortex, and a smaller total brain and grey matter volume. Both FA16:0 and FA16:1 were borderline significantly associated with a smaller white matter volume as well. The associations before adjustment largely echoed these results, but were much stronger (Figs. 4A and 5A).

Secondly, an increase in the relative concentration of several 18-carbon carrying composite measures was significantly associated with cortical thickness, and total brain, grey matter, and white matter volume after adjustment (23, 24, 21 and two out of a total of 54 18-carbon carrying composite measures, respectively) (Fig. 4B). The majority of the significantly associated measures (between 57% and 100%) were associated with a thicker cortex, and larger total brain, grey matter, and white matter volume. Lipids that were associated in the opposite direction – i.e. with smaller volumes or a thinner cortex – almost exclusively belonged to the sphingolipids (sphingomyelins, ceramides, dihydrosylceramides, lactosylceramides, hexosylceramides). Regarding the total fatty acid composite measures (Fig. 5B), we observed that total FA18:2 was associated with a thicker cortex and larger total brain and grey matter volume, total FA18:3 with a larger total brain and grey matter volume at an FDR-adjusted p-value and nominally with a larger white matter volume, and FA18:0 with a larger total brain and grey matter volume at a nominal significance level. In contrast, FA18:4 was nominally significantly associated with a thinner cortex. Before adjustment, the majority of the 18-carbon carrying composite measures were associated with a thinner cortex and smaller brain volumes (Fig. 4A). However, similar to the adjusted estimates, total FA18:2 was strongly associated with a thicker cortex and a larger total, grey matter and white matter volume (Fig. 5A).

Thirdly, we observed that an increase in the relative concentration of almost all 15-carbon and 17-carbon carrying lipids was significantly associated with a thicker cortex and larger total brain and grey matter volume after adjustment for sex, age, LDL-C, and HDL-C (Fig. 4B). This was supported by the results from the total fatty acid composite measures, where an increase in the relative concentration of both total FA15:0 and FA17:0 was associated with a thicker cortex as well as a larger total brain and grey matter volume (Fig. 5B). Unadjusted associations were similar for 15-carbon carrying lipids, but not 17-carbon carrying lipids (Figs. 4A and 5A).

Lastly, the majority of lipids carrying shorter fatty acids were associated with a thicker cortex and larger total brain, grey matter and white matter volume after, but not before, adjustment for sex, age, LDL-C, and HDL-C (Fig. 4A and B). The total fatty acid composites showed largely similar results as the class specific composite measures, as we observed that concentrations of FA12:0 and FA14:0 were significantly associated with a larger grey matter volume, while FA12:0, FA14:0 and FA14:1 were all borderline non-significantly associated with a larger total brain and grey matter volume (Fig. 5B).

**Fig. 6:**
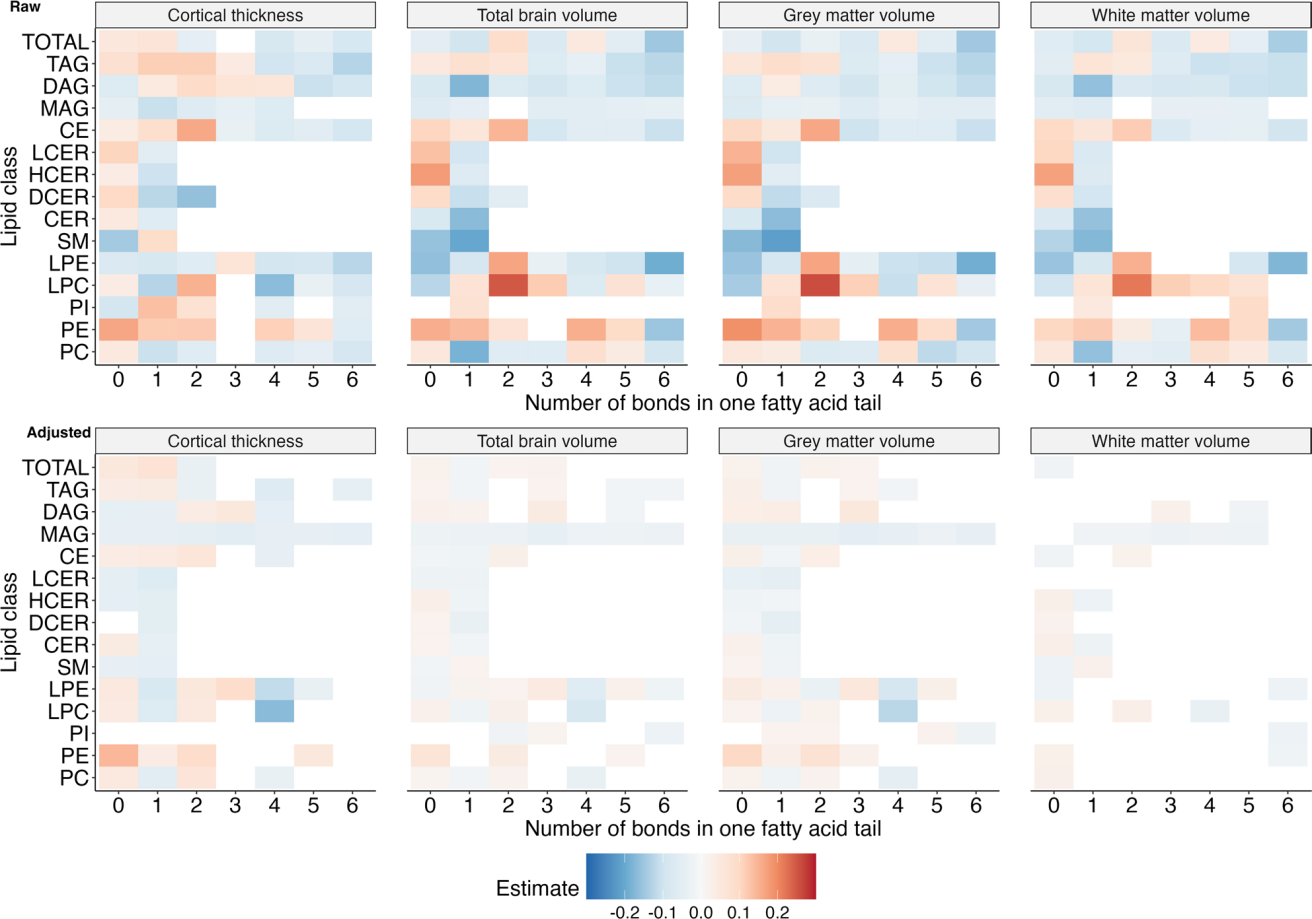
Associations between fatty acid composites and cortical thickness and brain volumes by degree of saturation. Significant raw (**A**) and adjusted (**B**) beta estimates for the relative concentration of composite measures per lipid class (y-axis) by the number of double bonds in the fatty acid tail (x-axis). Estimates were adjusted for age, sex, low-density lipoprotein cholesterol (LDL-C) and high-density lipoprotein cholesterol (HDL-C) concentrations and estimated intracranial volume where applicable

Besides the length of the fatty acid tail, we also found that the number of double bonds was important in determining a lipid’s association with cortical thickness and brain volumes (Fig. 6A and B). Specifically, out of the 54 highly polyunsaturated (> 4 double bonds) fatty acid composite measures, 16 were associated with cortical thickness, 17 with total brain volume, 14 with grey matter volume and 3 with white matter volume after adjustment for sex, age, LDL-C, and HDL-C. In almost all instances (86–100%), higher relative concentrations of the lipid composite measures were associated with a thinner cortex and smaller total brain, grey matter and white matter volume. This was further supported by the total fatty acid composites, where we found that the most highly unsaturated fatty acid (FA22:6) was borderline non-significantly associated with a smaller cortex and smaller total brain and white matter volume (Fig. 5B). In an enrichment analysis, where we compared the expected versus the observed number of significant associations, we found that saturated fatty acids were usually enriched compared to PUFAs and monounsaturated fatty acids, particularly in relation to white matter volume (Additional file 6). Before adjustment, most (57–100%) highly polyunsaturated fatty acid composite measures and total FA22:6 were similarly associated with a thinner cortex and smaller total brain, grey matter, and white matter volume (Figs. 4A and 5A).

An overview of the all associations between fatty acid composite measures before and after adjustment for sex, age, LDL-C, and HDL-C can be found in Additional file 5. Neither excluding outliers in the lipid measurements, nor excluding people on lipid-lowering medication substantially changed the aforementioned patterns (Additional file 5).

### Lipids are associated with different cortical regions

As the fatty acid composition was found to be a major factor in a lipid’s relation with cortical thickness, we explored whether these associations were uniform across the cortex or whether there were regional differences. Thus, for the relative concentrations of the 6 most significantly associated total fatty acids (FA15:0, FA16:0, FA16:1, FA18:2, FA22:0 and FA22:1) we performed a vertex-wide analysis, adjusting for sex, age, LDL-C and HDL-C.

**Fig. 7:**
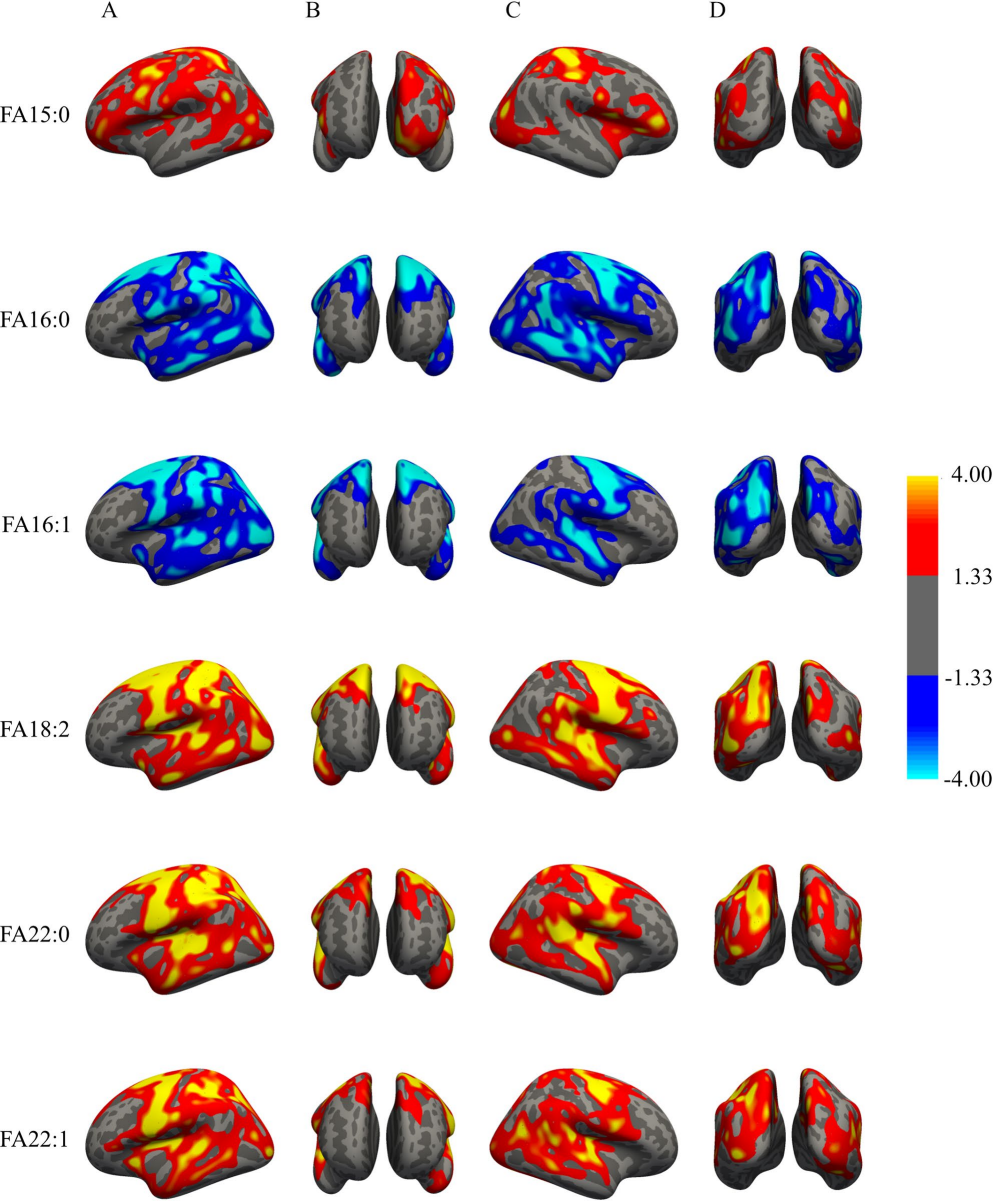
Cortex-wide associations between lipid composite measures and cortical thickness. Vertex-wide analysis results for the association between cortical thickness and the relative concentrations of total FA15:0, FA16:0, FA16:1, FA18:2, FA22:0, and FA22:1 at a cluster-corrected *p* value of < 0.05 from the left (**A**), frontal (**B**), right (**C**), and dorsal (**D**) view

As shown in Fig. 7, fatty acid composite measures were associated with different cortical regions in different, yet partly overlapping, patterns. In the left hemisphere, higher levels of FA15:0 were associated with a thicker pre-motor, primary motor, and primary somatosensory cortex as well as the ventromedial and ventrolateral cortex of the frontal lobe. In the right hemisphere, higher levels of FA15:0 were mainly linked to a thicker posterior parietal cortex and temporal lobe. In contrast, higher levels of FA16:0 were associated with a thinner cortex in almost all regions of both hemispheres, except the pre-frontal cortex. FA16:1 was most strongly associated with a thinner pre-motor, primary motor, and temporal lobe cortex in both hemispheres, as well as with a thinner somatosensory cortex and parietal lobe of the left hemisphere. Lastly, higher levels of FA18:2, FA22:0 and FA22:1 were similarly associated with a thicker cortex of the pre-motor cortex, primary motor cortex, and the temporal and parietal lobes. These associations were stronger in the left compared to the right hemisphere.

## Discussion

We related blood lipids to global brain volumetric and cortical thickness measures in a population-based study after adjustment for sex, age, LDL-C and HDL-C. Associations for lipid profiles – concentrations of lipids relative to each other – were generally more consistent and robust than the absolute concentrations of single lipids, emphasizing the relevance of lipid profiles rather than individual species. We mostly showed intraclass variability, which could partially be explained by differences in lipid structure, namely, chain length and degree of saturation. Specifically, higher relative concentrations of odd-chained and 18-carbon fatty acid carrying lipids were usually associated with a thicker cortex and a larger total brain and grey matter volume after adjustment for sex, age, LDL-C, and HDL-C, indicating better brain health. Lipids carrying shorter fatty acids, such as FA12:0 and FA14:0, were similarly associated. Conversely, most 16-carbon or PUFA (> 4 bonds) carrying lipids were associated with a smaller total brain, grey matter, and white matter volume, as well as a thinner cortex. Lastly, lipids were generally most strongly associated with the precentral gyrus and premotor cortex, but not the rest of the pre-frontal cortex.

While we observed certain class-dependent relationships for absolute lipid species levels with brain measures, such as triacylglycerols mostly being associated with a thinner cortex and phosphatidylethahnolamines with a thinner cortex and smaller brain volumes, the predominant pattern was one of intra-class variability. Even for lipid classes where the majority of species were associated with either better or worse cortical thickness and brain volumes, the few lipids associated in the opposite direction typically had a larger effect estimate. Intra-class variability in biological effects has been reported across various pathologies [11, 12, 15, 26, 37], including progression to AD [27]. For example, higher concentrations of some sphingomyelins were associated with a lower DM2 and CVD risk (SM(22:1), SM(22:0) and SM(20:0)) [16], whereas others were associated with a higher risk of these conditions (e.g. SM(26:1), SM(26:0), SM(24:1), SM(18:0), SM(16:0), SM(14:0)) [7, 16]. Similarly, distinct cholesteryl esters, phosphatidylcholines, phosphatidylethanolamines, lysophosphatidylcholines and lysophosphatidylethanolamines were linked to more favorable outcomes, including longevity (e.g. CE(20:5), CE(22:5), LPC(18:1), LPE(24:1), PC(40:5), PE(33:3), PE(34:2)) [15] and a lower CVD risk or severity (e.g. ether-linked phospholipids, LPC(17:0), LPC(18:2)) [7, 38], whereas others were associated with detrimental outcomes, namely an increased CVD (e.g. CE(16:1), PE(36:5), PC(32:0)) [7, 26, 38], and neurodegeneration (e.g. LPC(22:6), LPC(16:0), ether-linked lipids) risk [19, 27, 37, 38]. Ceramides reportedly have differential effects depending on their tail: where CER(16:0), CER(18:0) and CER(24:1) may increase CVD risk, CER(22:0) and CER(24:0) may confer a lower CVD and dementia risk [7, 24]. Even triacylglycerols, whose total concentration is a well-established CVD risk factor, seem to have different effects depending on their exact chemical composition [12, 18, 39]. We found similar patterns of intra-class variation in relation to cortical thickness and brain volumes, and expanded on previous studies by identifying inter-class patterns.

Across classes, length and degree of saturation of the fatty acid tail(s) were important in the association between lipids and brain structural measures. We found that 18-carbon carrying lipids were related to larger brain volumes and a thicker cortex. This supports, and expands, findings from a previous study associating 18-carbon carrying triacylglycerols with a thicker cortex [18]. Furthermore, 16-carbon carrying lipids were related to smaller volumes and a thinner cortex, which aligns with previous research linking higher levels of some 16-carbon carrying lipids to diseases, such as CE16:1 with COPD and CVD [7], and FA16:0 with DM2 [16]. Interestingly, 16- and 18-carbon carrying lipids are closely related in lipogenesis, as FA16 is converted in FA18 in the presence of elongase of very long chain fatty acids 6 (elovl6) [2]. Although our data cannot directly assess enzymatic activity, these observations suggest that elovl6 regulation could be interesting as a potential target for future research on improving brain health.

In this cross-sectional analysis, our results furthermore suggest that a lipidome with lower total PUFA and higher odd-chained fatty acid concentrations was related to a greater cortical thickness and brain volumes. Importantly, PUFAs are subdivided based on the first double bond placement in the fatty acid tail, where n-3, n-6 and n-9 PUFAs have a double bond starting at position 3, 6 and 9 from the methyl end, respectively [6, 40]. These subtypes can have antagonistic effects, e.g. decreasing (n-3) or increasing (n-6) CVD risk [41]. PUFAs are mainly dietary-derived: n-3 PUFAs primarily from fish and seafood and n-6 PUFAs from oils and foods of animal origin [42]. We could not distinguish between subtypes, as the Metabolon platform classifies PUFAs based on the number, but not the placement, of double bonds. Given that our study population mostly adheres to a typical western diet, characterized by a high n-6 to n-3 ratio [41], measured PUFAs likely represent mainly n-6 variants. However, this cannot be confirmed within the current dataset. While PUFAs are considered to be advantageous through increasing or upholding membrane fluidity [43], highly polyunsaturated fatty acids are also more susceptible to oxidative damage: every added double bond increases its sensitivity to oxidative damage. This may promote inflammatory processes and thereby the risk of neuroinflammation [21]. This potential vulnerability combined with the assumption that total PUFA concentrations likely represent the less advantageous n-6 variant in our population, may contribute to the observed detrimental relation between PUFAs and cortical thickness and brain volumes. Interestingly, odd-chained fatty acids are hypothesized to similarly increase membrane fluidity, but not the sensitivity to oxidative damage [21]. Odd-chained fatty acids are largely derived from dairy products [22, 44], and have been inversely associated with different pathologies, such as DM2 [17, 21]. We found that this beneficial effect might also apply to the brain. The beneficial effects of high odd-chained and low PUFA concentrations on brain volume and cortical thickness measures suggest that diet may influence brain morphology through the lipidome. However, more in-depth and longitudinal studies, particularly distinguishing between PUFA subtypes, are necessary to further understand the mechanisms driving these results.

When considering region-specific effects of 6 fatty acid composite measures, we found they were mainly associated with cortical regions outside of the pre-frontal cortex and affected the hemispheres asymmetrically. FA15:0 was most strongly associated with parietal lobe thickness, particularly in the right hemisphere, which is crucial for sensory input integration and spatial orientation [45]. FA18:2, FA22:0 and FA22:1 were associated with a thicker temporal and parietal lobe cortex, as well as the primary motor and pre-motor cortex. Parietal and temporal lobe damage is common in AD, and thinning of these regions precedes disease [46]. This suggests that maintaining levels of FA15:0 through dietary intake or sustaining higher levels of FA18:2, FA22:0 and FA22:1 through targeted interventions could be of particular interest in neurodegeneration. FA16:0 and FA16:1 were related to a thinner cortex of the temporal lobes, which are vital for sensory input processing and language comprehension, as well as the primary motor and premotor cortex, which are involved in movement [45]. Thinning of the premotor and motor cortex was previously observed in patients with motor neuron disease and associated with the level of disability [47]. Although our results suggest region-specific lipid needs, it is important to confirm these findings with longitudinal data.

Despite lipids comprising between 49 and 66% of white matter [48], we found few lipid associations with white matter volume after adjustment for sex, age, LDL-C, and HDL-C. This could have several explanations. Firstly, it might indicate that maintaining white matter volume is not dependent on a specific lipid species, but rather adequate lipid concentrations of any type. Secondly, white matter volume is a global measure and might not be sensitive enough to pick up small changes. More specific assessment of the white matter microstructure, as can be obtained e.g. with diffusion-weighted MRI, might be able to provide more information. Indeed, diffusion tensor imaging studies have reported plasma lipids to be associated with white matter microstructural alterations in both neurodegeneration and healthy aging [49, 50]. Recent work applying advanced dMRI-based microstructural modeling shows that neuronal density is reduced in patients with dyslipidemia while no changes in white matter volume measures are observed [51]. These findings complement our results and suggest a limited sensitivity of white matter volume measures to fully capture lipid-related alterations in the brain white matter. Future work should thus incorporate diffusion MRI-based metrics to further investigate the potentially subtle lipid-related microstructural changes in the brain white matter. Before adjustment, we found that many lipids related to a smaller white matter volume, which might be on account of age-related lipid breakdown in the brain.

The reported associations with white matter were largely in line with previous studies. In this work, we showed associations between white matter volume and specific lipids (i.e. SM, CERs, some phospholipids) with high enrichment, reflecting the natural lipid composition of the brain white matter. To determine the contribution of specific plasma lipid profiles to more subtle and early microstructural changes in the white matter, future investigations should incorporate diffusion MRI-derived metrics. Advanced diffusion MRI has been found to sensitively detect early white matter changes that typically precede gross volumetric white matter alterations both in large-scale cross-sectional and longitudinal studies [52–55].

When comparing the results for cortical thickness and grey matter volume, we found more and stronger associations with cortical thickness. Both cortical thickness and grey matter volume capture aspects of cerebral grey matter morphometry. However, while grey matter volume is a function of cortical thickness, it also depends on cortical surface area [56]. Cortical surface area and cortical thickness are independent, both globally and regionally, and genetically uncorrelated [56, 57]. Our finding of different relations between lipid concentrations and cortical thickness and grey matter volume suggests lipidomic differences across cortical regions and aspects of brain cortical structure, which is further supported by our vertex-wide analysis. Research detailing lipid-related mechanisms with specific brain imaging features is warranted.

A strength of our study is that we assessed a wide number of lipid species and fatty acid composition across multiple lipid classes, which, to the best of our knowledge, has not been done before at this scale and in a general population sample. This enabled us to provide more details on lipids across classes. Moreover, the study design and inclusion of different imaging markers allowed us to elucidate mechanisms of general brain physiology, rather than being specific to a single disease or disorder. A possible limitation is that our participants are relatively healthy and well-educated. As such, the cortical thickness or brain volumes might not (yet) be as strongly affected as would be expected in a more diverse population. To the extent that this has restricted the variability in our sample, it may have limited our power to find even more significant effects. However, we consider it unlikely that this would have resulted in spurious findings. Moreover, our study was conducted in Bonn, Germany, and in line with the local population composition, our participants were mostly of western European descent. Thus, generalizability to persons from other ancestries remains unknown. Furthermore, we could not assess the temporality of the associations between lipids and the brain. Although observed blood lipid patterns could be causally related to cortical thickness and brain volumes, confirmation in longitudinal studies is required. After all, breakdown of lipids within the brain could also directly influence the peripheral lipid profile: it is possible that the circulating lipidome reflects the observed damage, rather than causing it. Understanding temporality is especially important for possible practical applications of lipids. While our results suggest, for example, that dairy products might be beneficial, follow-up time is necessary before lipid levels can be manipulated to enhance brain health or improve longevity. Relatedly, while measuring lipids in plasma is less invasive than measuring it directly in the brain or cerebrospinal fluid, it has to be noted that it is not a direct reflection of the brain lipidome. This ought to be considered in future research as well. Last, certain lipid species and lipid classes might be strongly correlated, which could mean that observed associations for multiple lipids are driven by a singular lipid. This could be the case for some of the associations found for triacylglycerols and diacylglycerols, as these tend to be strongly related. However, a large part of our lipids is not strongly correlated, as our lipid panel spans multiple lipid classes that do not share a (genetic) basis. Regardless of a shared background or relatedness, we also show that the whole lipid profile – i.e. the relative concentrations – might be crucial to consider in future research. After all, we found more numerous and stronger relations between relative lipid concentrations and the brain. This implies that these lipids work in tandem with each other and that the effect of one lipid might be dependent on the concentrations of another. This is also important in light of the relatively small effect estimates. Most omics analyses show small effect sizes for single molecular markers (e.g. genome-wide association analyses (GWAS)). However, this does not necessarily diminish the relevance of the effects. Indeed, while single lipids are unlikely to emerge as important prediction biomarkers, even small effect sizes may point to important etiologic mechanisms. As for risk prediction, it might be useful to estimate the joint effect of multiple lipids in the form of a lipid profile. Akin to polygenic risk scores, this might both increase the effect size and account for their relatedness. Lastly, our study was mostly concerned with the associations between blood lipid levels and the brain – regardless of what determined these lipid profiles. In future research, it would be interesting to further investigate these determinants. After all, lipids may be dietary-derived [16, 41], but are also influenced by other factors such as physical activity [58], toxins in the environment [59], and genetics [60]. Further adjustment for these factors, or investigating these possible influences on the lipid profile as a whole, could further improve our understanding of the role of lipids in health and disease – and how to modify them to improve outcomes.

## Conclusions

We showed that biochemical features of circulating lipids are crucial in their relation with cortical thickness and brain volumes. Specifically, odd-chained and 18-carbon carrying lipids seem to be beneficial, while 16-carbon carrying and highly unsaturated lipids seem to be detrimental. Our research highlights potential targets for future research, aimed at identifying biomarkers and developing (preventive) therapeutics. We also emphasize that the lipid profile, rather than absolute concentrations of specific lipids, is crucial to investigate.

## Supplementary Information

Supplementary Material 1.

Supplementary Material 2.

Supplementary Material 3.

Supplementary Material 4.

Supplementary Material 5.

Supplementary Material 6.

## Data Availability

The data for this manuscript are not publicly available due to data protection regulations. Access to data can be provided to scientists in accordance with the Rhineland Study’s Data Use and Access Policy. Requests for additional information and/or access to the datasets can be send to RS-DUAC@dzne.de.All authors had full access to all of the data in the study and take responsibility for the integrity of the data and the accuracy of the data analysis.The code has been made publicly available (https://github.com/EN-Landstra/Plasma-Lipids-And-Brain).

## Abbreviation

AD: Alzheimer’s disease
BUME: butanol/methanol
COPD: Chronic obstructive pulmonary disorder
CVD: Cardiovascular disease
DM2: Type 2 diabetes
FDR: False discovery rate
FWHM: Full-width-half-maximum
GWAS: Genome-wide association analysis
HDL-C: High-density lipoprotein cholesterol
LDL-C: Low-density lipoprotein cholesterol
MRI: Magnetic resonance imaging
PUFA: Polyunsaturated fatty acid
