# Supplementary material for: Plasma complex lipids in relation to cortical thickness and brain volumes: results from the population-based Rhineland study
Source: Lipids Health Dis. 2026 Mar 19;25:104. doi: 10.1186/s12944-026-02930-5 (PMC13063565; doi:10.1186/s12944-026-02930-5)
Supplement: Supplementary file 1 — Supplementary Material 1. [file 12944_2026_2930_MOESM1_ESM.docx]

**Additional file 1:** Overview of the mean. standard deviation (SD) and range of lipid concentrations for participants with available MRI data (n= 3,248)

| Lipid class | Concentration | Mean (SD) | Minimum - maximum |
| --- | --- | --- | --- |
| *CE* | nmol/mL | 2,872.01 (615.16) | 56.38 - 10,408.17 |
|  | mol% | 41.72 (3.98) | 2.99 - 62.67 |
| *CER* | nmol/mL | 5.41 (1.39) | 1.68 - 15.74 |
|  | mol% | 0.08 (0.02) | 0.03 - 0.47 |
| *DAG* | nmol/mL | 25.00 (16.01) | 1.63 - 245.32 |
|  | mol% | 0.35 (0.14) | 0.09 - 1.37 |
| *DCER* | nmol/mL | 1.41 (0.50) | 0.40 - 10.51 |
|  | mol% | 0.02 (0.01) | 0.01 - 0.12 |
| *HCER* | nmol/mL | 4.36 (1.10) | 0.86 - 11.68 |
|  | mol% | 0.06 (0.02) | 0.02 - 0.17 |
| *LCER* | nmol/mL | 3.33 (0.73) | 1.42 - 8.23 |
|  | mol% | 0.05 (0.01) | 0.01 - 0.12 |
| *LPC* | nmol/mL | 173.68 (39.34) | 18.00 - 364.36 |
|  | mol% | 2.57 (0.61) | 0.56 - 5.39 |
| *LPE* | nmol/mL | 6.32 (1.85) | 1.86 - 17.16 |
|  | mol% | 0.09 (0.03) | 0.03 - 0.23 |
| *MAG* | nmol/mL | 2.35 (4.73) | 0.12 - 126.92 |
|  | mol% | 0.04 (0.07) | 0.00 - 1.49 |
| *PC* | nmol/mL | 2,022.49 (383.70) | 509.32 - 3,814.27 |
|  | mol% | 29.53 (3.12) | 16.72 - 52.51 |
| *PE* | nmol/mL | 157.26 (43.28) | 33.86 - 872.98 |
|  | mol% | 2.30 (0.90) | 0.39 - 46.33 |
| *PI* | nmol/mL | 32.35 (8.14) | 4.47 - 71.41 |
|  | mol% | 0.47 (0.09) | 0.05 - 0.93 |
| *SM* | nmol/mL | 490.95 (89.03) | 200.85 - 952.34 |
|  | mol% | 7.23 (1.09) | 2.68 - 19.87 |
| *TAG* | nmol/mL | 1,121.76 (716.03) | 20.44 - 9,634.71 |
|  | mol% | 15.49 (6.13) | 1.09 - 54.11 |
| *Abbreviations: CE (Cholesteryl ester), CER (Ceramide), DAG (Diacylglycerol), DCER (Dihydrosylceramides), HCER (Hectosylceramides), LCER (Lactosylceramides), LPC (Lysophosphatidylcholine), LPE (Lysophosphatidylethanolamine), MAG (Monoacylglycerol), PC (Phosphatidylcholine), PE (Phosphatidylethanolamine), PI (Phosphatidylinositol), SD (Standard deviation), SM (Sphingomyelin), TAG (Triacylglycerol)* | | | |
