# Supplementary material for: Plasma complex lipids in relation to cortical thickness and brain volumes: results from the population-based Rhineland study
Source: Lipids Health Dis. 2026 Mar 19;25:104. doi: 10.1186/s12944-026-02930-5 (PMC13063565; doi:10.1186/s12944-026-02930-5)
Supplement: Supplementary file 2 — Supplementary Material 2. [file 12944_2026_2930_MOESM2_ESM.docx]

**Additional file 2:** Overview of lipid nomenclature

| Lipid categories  (n= 3) | Lipid classes (n= 14) | *Species (n= 944)** | | *Fatty acid composite measures*  *(n= 267)** | |
| --- | --- | --- | --- | --- | --- |
|  |  | *Number (%)* | *Example* | *Number (%)* | *Example* |
| Neutral lipids | Triacylglycerol | 518 (54.9%) | TAG50:1(FA20:3) | 21 (7.9%) | TAG(FA12:0) |
|  | Diacylglycerol | 58 (6.1%) | DAG(16:1/20:0) | 19 (7.1%) | DAG(FA22:5) |
|  | Monoacylglycerol | 26 (2.8%) | MAG(18:3) | Same as species | |
|  | Cholesteryl ester | 26 (2.8%) | CE(12:0) | Same as species | |
| Phospholipids | Phosphatidylcholine | 101 (10.7%) | PC(15:0/22:0) | 23 (8.6%) | PC(FA17:0) |
|  | Phosphatidylethanolamine** | 94 (10.0%) | PE(18:1/18:1) | 16 (6.0%) | PE(FA16:1) |
|  | Phosphatidylinositol | 26 (2.8%) | PI(18:1/18:2) | 13 (4.9%) | PI(FA20:3) |
|  | Lysophosphatidylcholine | 18 (1.9%) | LPC(14:0) | Same as species | |
|  | Lysophospathdiylethanolamine | 16 (1.7%) | LPE(18:1) | Same as species | |
| Sphingolipids | Sphingomyelin | 12 (1.3%) | SM(26:0) | Same as species | |
|  | Ceramide | 12 (1.3%) | CER(20:1) | Same as species | |
|  | Dihydosylceramide | 13 (1.4%) | DCER(22:2) | Same as species | |
|  | Hexosylceramide | 12 (1.3%) | HCER(18:0) | Same as species | |
|  | Lactosylceramide | 12 (1.3%) | LCER(16:0) | Same as species | |

**Lipid species are defined by complete information on the total number of carbons and double bonds, whereas fatty acid composite measures sum up the concentration of all lipids within a class of a specific length and degree of saturation. In case of one-tailed lipids, the fatty acid composite measure is thus equal to the species. In addition, we have information on 28 total fatty acid composite measures, which sum up the concentration of a specific fatty acid tail across all lipid classes.
**Containing phosphatidylethanolamine esters (n=41), phosphatidylethanolamine ethers (n= 17) and phosphatidylethanolamine plasmalogens (n= 36)*
