# Supplementary material for: Plasma complex lipids in relation to cortical thickness and brain volumes: results from the population-based Rhineland study
Source: Lipids Health Dis. 2026 Mar 19;25:104. doi: 10.1186/s12944-026-02930-5 (PMC13063565; doi:10.1186/s12944-026-02930-5)
Supplement: Supplementary file 3 — Supplementary Material 3. [file 12944_2026_2930_MOESM3_ESM.docx]

**Additional file 3:** Overview of the number of significant lipid species concentrations per lipid class and outcome

| Class | Species | Cortical thickness | | Total brain volume | | Grey matter volume | | White matter volume | |
| --- | --- | --- | --- | --- | --- | --- | --- | --- | --- |
| *Absolute concentrations (nmol)* |  | *Negative (n= 766)* | *Positive  (n= 25)* | *Negative  (n= 365)* | *Positive  (n= 161)* | *Negative  (n= 430)* | *Positive  (n= 121)* | *Negative  (n= 262)* | *Positive  (n= 228)* |
| Monoacylglycerol | 26 | 8 | 2 | 4 | 1 | 6 | 1 | 2 | - |
| Diacylglycerol | 58 | 53 | 1 | 16 | 11 | 27 | 6 | 10 | 15 |
| Triacylglycerol | 518 | 483 | - | 110 | 130 | 151 | 94 | 45 | 192 |
| Cholesteryl ester | 26 | 20 | - | 22 | - | 23 | - | 18 | - |
| Phosphatidylcholine | 101 | 70 | 7 | 75 | 1 | 79 | 1 | 67 | 2 |
| *Phosphatidylethanolamine* | 94 | 63 | 3 | 67 | 1 | 69 | 1 | 61 | - |
| Phosphatidylinositol | 26 | 8 | 4 | 14 | - | 15 | - | 14 | - |
| Lysophosphatidylethanolamine | 16 | 11 | 1 | 5 | 2 | 7 | 2 | 3 | 3 |
| Lysophosphatidylcholine | 18 | 5 | 3 | - | 13 | 1 | 14 | - | 14 |
| Ceramide | 12 | 11 | - | 11 | - | 12 | - | 9 | - |
| Dihydroceramide | 13 | 11 | - | 11 | - | 11 | - | 8 | - |
| Hexosylceramide | 12 | 8 | 1 | 9 | 2 | 9 | 2 | 7 | 2 |
| Lactosylceramide | 12 | 5 | 3 | 9 | - | 8 | - | 6 | - |
| Sphingomyelin | 12 | 10 | - | 12 | - | 12 | - | 12 | - |
| *Relative concentrations (mol%)* |  | *Negative  (n= 370)* | *Positive  (n= 215)* | *Negative  (n= 363)* | *Positive  (n= 190)* | *Negative  (n= 356)* | *Positive  (n= 164)* | *Negative  (n= 338)* | *Positive  (n= 164)* |
| Monoacylglycerol | 26 | 8 | 1 | 10 | 1 | 15 | 1 | 5 | - |
| Diacylglycerol | 58 | 26 | 13 | 32 | 9 | 31 | 11 | 32 | 7 |
| Triacylglycerol | 518 | 232 | 111 | 217 | 90 | 217 | 99 | 211 | 67 |
| Cholesteryl ester | 26 | 13 | 5 | 11 | 7 | 11 | 8 | 10 | 7 |
| Phosphatidylcholine | 101 | 33 | 20 | 25 | 15 | 27 | 20 | 22 | 19 |
| *Phosphatidylethanolamine* | 94 | 10 | 39 | 12 | 47 | 3 | 4 | 11 | 46 |
| Phosphatidylinositol | 26 | 4 | 5 | 5 | 4 | 9 | 2 | 4 | 2 |
| Lysophosphatidylethanolamine | 16 | 10 | 4 | 8 | 2 | 9 | 5 | 7 | 2 |
| Lysophosphatidylcholine | 18 | 6 | 4 | 10 | 5 | 6 | 2 | 6 | 5 |
| Ceramide | 12 | 5 | 3 | 7 | 2 | 3 | 1 | 8 | 2 |
| Dihydroceramide | 13 | 3 | 2 | 3 | 1 | 10 | 2 | 2 | 1 |
| Hexosylceramide | 12 | 8 | 2 | 10 | 2 | 8 | 2 | 10 | 2 |
| Lactosylceramide | 12 | 8 | 2 | 9 | 2 | 4 | 3 | 6 | 1 |
| Sphingomyelin | 12 | 4 | 4 | 4 | 3 | 3 | 4 | 4 | 3 |
