# Supplementary material for: Plasma complex lipids in relation to cortical thickness and brain volumes: results from the population-based Rhineland study
Source: Lipids Health Dis. 2026 Mar 19;25:104. doi: 10.1186/s12944-026-02930-5 (PMC13063565; doi:10.1186/s12944-026-02930-5)
Supplement: Supplementary file 4 — Supplementary Material 4. [file 12944_2026_2930_MOESM4_ESM.docx]

**Additional file 4:** Overview of the number of significant lipid species concentrations per lipid class and outcome after adjustment for sex, age, LDL-C and HDL-C

| Class | Species | Cortical thickness | | Total brain volume | | Grey matter volume | | White matter volume | |
| --- | --- | --- | --- | --- | --- | --- | --- | --- | --- |
| *Absolute concentrations (nmol)* |  | *Negative (n= 391)* | *Positive  (n= 25)* | *Negative  (n= 78)* | *Positive  (n= 70)* | *Negative  (n= 100)* | *Positive  (n= 98)* | *Negative  (n= 0)* | *Positive  (n= 5)* |
| Monoacylglycerol | 26 | - | 3 | - | 4 | - | 4 | - | - |
| Diacylglycerol | 58 | 35 | 2 | 1 | 4 | 1 | 7 | - | - |
| Triacylglycerol | 518 | 239 | - | 18 | 29 | 18 | 56 | - | - |
| Cholesteryl ester | 26 | 5 | - | 1 | - | 1 | 2 | - | - |
| Phosphatidylcholine | 101 | 40 | 4 | 32 | 5 | 43 | 4 | - | - |
| Phosphatidylethanolamine | 94 | 46 | 4 | 13 | 7 | 19 | 9 | - | - |
| Phosphatidylinositol | 26 | 1 | 2 | 1 | - | 1 | 1 |  | - |
| Lysophosphatidylethanolamine | 16 | 7 | 3 | 3 | 6 | 3 | 5 | - | - |
| Lysophosphatidylcholine | 18 | 2 | 4 | 2 | 9 | 2 | 5 | - | 3 |
| Ceramide | 12 | 4 | 1 | - | 3 | - | 2 | - | 1 |
| Dihydroceramide | 13 | 2 | - | 3 | - | 5 | - | - | - |
| Hexosylceramide | 12 | 4 | 1 | 1 | - | 4 | 1 | - | - |
| Lactosylceramide | 12 | 3 | 1 | 3 | - | 3 | 1 | - | - |
| Sphingomyelin | 12 | 3 | - | - | 3 | - | 1 | - | 1 |
| *Relative concentrations (mol%)* |  | *Negative  (n= 167)* | *Positive  (n= 121)* | *Negative  (n= 132)* | *Positive  (n= 165)* | *Negative  (n= 143)* | *Positive  (n= 222)* | *Negative  (n= 11)* | *Positive  (n= 11)* |
| Monoacylglycerol | 26 | 14 | 3 | 20 | 3 | 22 | 3 | 2 | - |
| Diacylglycerol | 58 | 11 | 9 | 4 | 9 | 1 | 1 | - | 1 |
| Triacylglycerol | 518 | 89 | 50 | 46 | 100 | 38 | 149 | - | - |
| Cholesteryl ester | 26 | 2 | 7 | 2 | 3 | 2 | 5 | - | - |
| Phosphatidylcholine | 101 | 16 | 15 | 23 | 11 | 38 | 14 | - | 3 |
| *Phosphatidylethanolamine* | 94 | 7 | 19 | 6 | 17 | 12 | 18 | 1 | 1 |
| Phosphatidylinositol | 26 | 2 | 2 | 4 | 1 | 4 | 3 | - | - |
| Lysophosphatidylethanolamine | 16 | 4 | 4 | 5 | 6 | 4 | 5 | 2 | - |
| Lysophosphatidylcholine | 18 | 4 | 5 | 5 | 4 | 5 | 5 | 2 | 2 |
| Ceramide | 12 | 4 | 3 | 4 | 2 | 2 | 2 | 2 | 1 |
| Dihydroceramide | 13 | 2 | - | 5 | 1 | 5 | - | - | - |
| Hexosylceramide | 12 | 6 | 1 | 2 | 1 | 5 | 2 | 1 | 1 |
| Lactosylceramide | 12 | 3 | 1 | 3 | 3 | 3 | 1 | - | - |
| Sphingomyelin | 12 | 3 | 2 | 3 | 4 | 2 | 2 | 1 | 2 |
