# Supplementary material for: Plasma complex lipids in relation to cortical thickness and brain volumes: results from the population-based Rhineland study
Source: Lipids Health Dis. 2026 Mar 19;25:104. doi: 10.1186/s12944-026-02930-5 (PMC13063565; doi:10.1186/s12944-026-02930-5)
Supplement: Supplementary file 6 — Supplementary Material 6. [file 12944_2026_2930_MOESM6_ESM.docx]

**Additional file 6:** Enrichment of lipids depending on their degree of saturation. Enrichment is calculated based on the results after adjustment for sex, age, LDL-C and HDL-C

| Lipid saturation | Enrichment* | | | |
| --- | --- | --- | --- | --- |
|  | *Cortical thickness* | *Total brain volume* | *Grey matter volume* | *White matter volume* |
| Absolute concentrations (nmol) |  |  |  |  |
| *Saturated fatty acids* | 0.76 | 1.04 | 1.30 | 9.39 |
| *Monounsaturated fatty acids* | 0.92 | 0.98 | 1.12 | 1.26 |
| *Polyunsaturated fatty acids* | 1.22 | 0.99 | 0.79 | 0.11 |
| Relative concentrations (mol%) |  |  |  |  |
| *Saturated fatty acids* | 1.10 | 0.89 | 1.09 | 3.58 |
| *Monounsaturated fatty acids* | 1.04 | 1.07 | 1.22 | 0.80 |
| *Polyunsaturated fatty acids* | 0.93 | 1.02 | 0.83 | 0.43 |

***** Enrichment was calculated by comparing the number of observed and expected significant associations. A value of 1 indicates there are more observations then expected
